# Immune-checkpoint blockade and candidate predictive biomarkers in HIV-negative Kaposi sarcoma: current evidence and a practical research framework

**DOI:** 10.3389/fimmu.2026.1979969

**Published:** 2026-10-16

**Authors:** Yang Song, Yan Fang, Lin Zeng, Hua Guan

**Affiliations:** 1 Department of Oncology, Sichuan Academy of Medical Sciences and Sichuan Provincial People’s Hospital, Chengdu, China; 2 School of Medicine, University of Electronic Science and Technology of China, Chengdu, China

**Keywords:** HHV-8, HIV-negative, immune checkpoint inhibitor, Kaposi sarcoma, KSHV, narrative review, pembrolizumab, translational research

## Abstract

Kaposi sarcoma (KS) is a KSHV/HHV-8-driven vascular neoplasm with heterogeneous HIV-negative epidemiological settings. Immune-checkpoint blockade is biologically plausible, but direct clinical evidence remains limited to a small, non-comparative phase II cohort of pembrolizumab in classic or endemic KS. Its 71% best objective response rate is a signal that warrants confirmation, not comparative evidence or a basis for biomarker-guided treatment. This narrative review distinguishes established clinical observations from candidate translational hypotheses, appraises analytical and biological limitations of proposed markers, and outlines requirements for prospective subtype-stratified validation. PD-L1 expression, tumor mutational burden, and KSHV DNA should not be used as stand-alone selection or exclusion tests in routine care.

## 1 Introduction

Kaposi sarcoma is an angioproliferative neoplasm caused by Kaposi sarcoma-associated herpesvirus (KSHV), also designated human herpesvirus 8 (HHV-8) (1–3). Although epidemic HIV-associated KS remains the most familiar form, a substantial and biologically heterogeneous group arises in people without HIV (4–6). This group encompasses classic KS, endemic African KS, iatrogenic and transplant-associated KS, and cases that cannot be confidently assigned to a conventional epidemiological subtype. These categories differ in age, geography, immune context, natural history, visceral risk, and opportunities for immune restoration. Consequently, pooling all HIV-negative disease without stratification can obscure clinically meaningful treatment effects.

Local therapy is appropriate for limited disease, whereas disseminated, symptomatic, rapidly progressive, or visceral KS commonly requires systemic treatment (5, 7, 8). Liposomal anthracyclines, paclitaxel, interferon, immunomodulatory agents, and antiangiogenic approaches can be effective, but durable remission is not assured and cumulative toxicity is relevant (9–11). Because KS is both virus driven and shaped by immune dysregulation, immune-checkpoint inhibition has a plausible biological rationale (12–14). This review addresses three questions: what direct clinical evidence supports checkpoint blockade in HIV-negative KS; which biomarker observations are established versus hypothesis generating; and what prospective study features are required before a biomarker-guided strategy can be considered. It does not recommend a regimen for an individual patient or establish a validated treatment-selection test.

## 2 Narrative review methods and scope

This narrative review was prepared to contextualize direct clinical evidence for immune-checkpoint blockade in HIV-negative KS and to identify research priorities. Searches were last updated on July 25, 2026, in PubMed/MEDLINE and ClinicalTrials.gov. The search combined the concept groups (Kaposi sarcoma OR classic OR endemic OR iatrogenic) AND (HIV-negative OR non-HIV) AND (immune checkpoint inhibitor OR pembrolizumab OR nivolumab OR PD-1 OR PD-L1) AND (biomarker OR KSHV DNA OR HHV-8 DNA); reference lists of key studies and reviews were also examined. We included peer-reviewed clinical studies, cohorts, case series, mechanistic studies informative for biological plausibility, and registry records relevant to the development landscape. We excluded duplicate reports, retained the most complete report when publications overlapped, and did not use recruiting, withdrawn, terminated, status-unknown, or unpublished records as efficacy evidence. A narrative approach was appropriate because the direct evidence base is sparse, subtypes and host contexts are heterogeneous, and studies use non-comparable designs and assays. This was not a systematic review: no exhaustive multi-database search, PRISMA flow diagram, formal risk-of-bias assessment, or meta-analysis was undertaken.

Clinical studies, cohorts, case series, and informative reports were interpreted according to design, HIV-negative population relevance, response definition, follow-up, and peer-review status. Mechanistic studies were used to explain biological plausibility, not to establish clinical prediction. Candidate biomarkers were considered clinically credible only when their assay context was specified and a treatment-by-marker relationship was evaluated; simple associations with disease extent or response were not treated as predictive evidence. Registry entries without posted or published outcomes were used only to describe the development landscape. The literature assessment reported here was completed on July 25, 2026.

### 2.1 Scope, terminology, and evidence hierarchy

The expression HIV-negative KS is useful but insufficiently precise. It identifies the absence of a major acquired immunodeficiency state while leaving unresolved the epidemiological subtype, duration and intensity of other immune perturbations, and geographical context of KSHV exposure. Classic KS typically develops in older men from Mediterranean, Middle Eastern, or Eastern European populations and often follows an indolent cutaneous course. Endemic KS occurs in KSHV-endemic regions and includes phenotypes ranging from indolent adult disease to aggressive lymphadenopathic disease. Iatrogenic KS arises during pharmacological immune suppression, most conspicuously after solid-organ transplantation but also during treatment of autoimmune and inflammatory disorders. A further group includes younger patients outside conventional endemic settings, men who have sex with men without HIV, individuals with unrecognized primary immune defects, and patients whose available history does not permit reliable assignment. This review therefore uses HIV-negative as an umbrella term but preserves subtype labels whenever the primary report provides them. This distinction is not semantic. Age, immune reserve, lesion distribution, viral burden, treatment history, and the reversibility of immune suppression can all influence both checkpoint efficacy and immune-related toxicity.

Clinical efficacy evidence was ranked pragmatically. Prospective interventional studies with prespecified response criteria were considered the highest available level, followed by prospective cohorts, retrospective series, and individual reports. Mechanistic observations were not promoted to clinical predictors unless a treatment-by-biomarker relationship had been examined. A variable associated with disease extent or survival was classified as prognostic rather than predictive unless it differentially identified benefit from a particular therapy. This distinction is especially important for KSHV DNA, inflammatory markers, lymphocyte counts, and PD-L1, all of which can correlate with disease biology without necessarily predicting a causal benefit from PD-1 blockade. Registered studies were used to map the development landscape, not to infer activity. Trials listed as status unknown, withdrawn, terminated, or recruiting were described using those exact categories. Published results were linked to the corresponding registry identifier when possible, and HIV-associated datasets were treated as supportive biological or safety context rather than direct estimates for an HIV-negative population. The principal differences among these HIV-negative KS settings are summarized in **Table 1**.

**Table 1:** Comparative clinical and immune-context features of HIV-negative Kaposi sarcoma settings relevant to interpretation of checkpoint-inhibitor evidence.

Setting	Host/immune context	Typical pattern	Implication for ICI evidence
Classic	Older adults; immunosenescence may contribute	Often indolent cutaneous disease	Comorbidity and competing risks affect safety and interpretation
Endemic	KSHV-endemic geography; heterogeneous host context	Adult cutaneous through aggressive nodal phenotypes	Geographic representation and subtype-specific estimates are essential
Iatrogenic nontransplant	Drug-related immune suppression that may be partly reversible	Variable pace and sites	Modify immune suppression when feasible; avoid attributing benefit to ICI alone
Transplant-associated	Allograft-dependent immunosuppression	May improve after immunosuppression adjustment	Graft-rejection risk requires separate assessment; direct ICI evidence is insufficient

### 2.2 Sources of bias and anticipated heterogeneity

Several biases shape the literature. Referral and eligibility selection favor patients treated at specialist centers who have adequate performance status, measurable cutaneous disease, and capacity for serial visits or biopsies; aggressive visceral presentations, major organ dysfunction, frailty, and transplant recipients may therefore be underrepresented. Reporting and publication bias favor exceptional responses, unusual toxicity, or biologically striking PD-L1 findings, whereas primary resistance may be less visible. Outcome measurement is also vulnerable to error because multifocal lesions vary in color and thickness, and edema may improve independently of infected-cell clearance; older reports often omit duration of response, lesion counts, standardized photographs, or patient-reported outcomes. Finally, subtype mixing can obscure differences in host age, geography, immune background, tumor behavior, and treatment context. These limitations support a qualitative synthesis with explicit uncertainty rather than a pooled response proportion that appears more precise than the underlying evidence.

Biomarker studies carry additional risks. Archival tissue may be temporally mismatched to the treated lesion and cannot capture spatial variation across plaques, nodules, lymph nodes, and visceral sites. Preanalytical conditions, biopsy cellularity, specimen processing and storage, and assay quality control can further affect reproducibility. PD-L1 results vary by antibody clone, scoring algorithm, threshold, and cellular compartment; KSHV DNA depends on specimen type, collection timing, extraction, and assay calibration. Small datasets can select favorable cutoffs after observing outcomes, while multiple correlated immune measurements increase false-discovery risk. Host genetic and immunologic background, age and comorbidity, pharmacologic immunosuppression and its reversibility, prior therapy, geographic context, and possible KSHV strain variation may also contribute to response heterogeneity but are not established predictors. Accordingly, the framework emphasizes prespecified assays, locked thresholds, longitudinal sampling, transparent missing-data handling, reproducibility testing, and external validation.

### 2.3 Limitations of this review

This narrative review is limited by non-exhaustive database coverage, reliance on published and registry-accessible information, heterogeneous HIV-negative populations, inconsistent staging and response definitions, and variable biomarker specimen and assay methods. The available evidence cannot estimate comparative effectiveness or establish a predictive marker. These limitations are why the framework is intended to guide prospective research rather than routine treatment selection.

## 3 Biological rationale for immunotherapy

KSHV persists predominantly as a latent episome in tumor cells. Latency-associated nuclear antigen maintains the viral genome and influences host transcription, cell-cycle regulation, and apoptosis (15–17). Intermittent lytic activity and paracrine viral products contribute to cytokine production, endothelial reprogramming, vascular permeability, and angiogenesis (13, 18, 19). Viral proteins and noncoding RNAs engage NF-kB, PI3K-AKT-mTOR, JAK-STAT, and hypoxia-related pathways, creating a lesion that combines infected spindle cells with abundant inflammatory and vascular components (20–22).

Persistent viral infection should provide non-self antigens that are recognizable by T cells. KSHV nevertheless reduces antigen presentation, alters interferon signaling, recruits suppressive immune populations, and favors T-cell dysfunction. PD-1/PD-L1 signaling is one component of this immune-evasion network. Blocking PD-1 may therefore restore responses against viral and tumor-associated antigens even when the somatic mutation burden is low. This mechanism explains why negative PD-L1 staining or low tumor mutational burden cannot be assumed to exclude benefit. Conversely, lesions with severe immune exclusion, irreversible T-cell dysfunction, minimal antigen presentation, or dominant immunosuppressive myeloid programs may remain resistant. The proposed biologicalframework is summarized in **Figure 1**.

**Figure 1:**
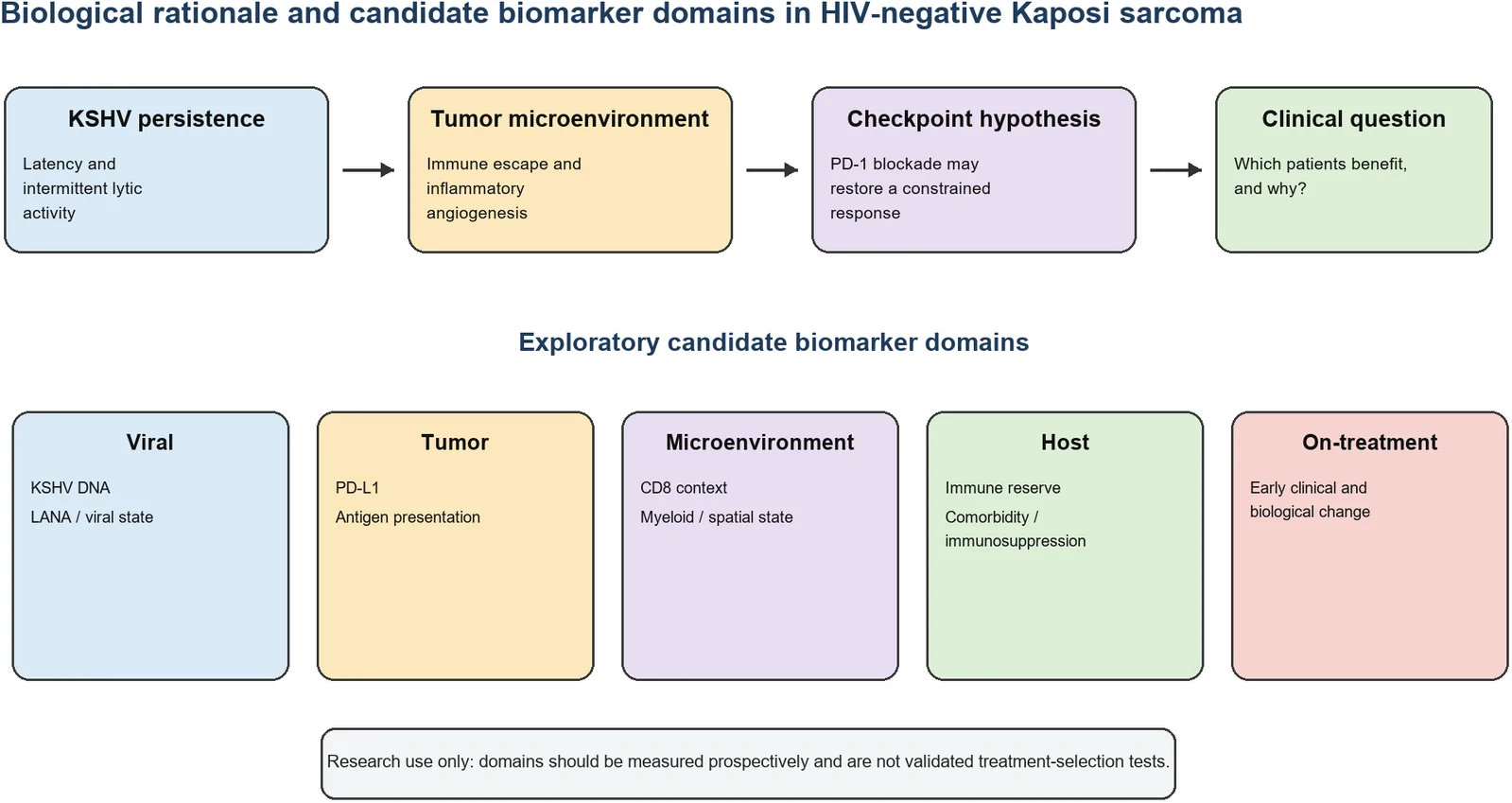
Simplified author-created framework linking KSHV persistence, tumor-microenvironment biology, checkpoint hypothesis, and the clinical question in HIV-negative Kaposi sarcoma. The lower panel lists five exploratory measurement domains: viral, tumor, microenvironment, host, and on-treatment. The image is intended to organize prospective research questions; it does not imply that any single marker or composite model is ready for treatment selection. LANA, latency-associated nuclear antigen; KSHV, Kaposi sarcoma-associated herpesvirus.

### 3.1 KSHV entry, endothelial reprogramming, and lineage plasticity

KSHV infects endothelial and other permissive cells through interactions involving heparan sulfate, integrins, and additional entry factors. Infection is followed by extensive transcriptional and epigenetic reprogramming. The spindle cell, the defining neoplastic cell of KS, expresses endothelial markers but frequently displays mixed blood-vascular and lymphatic characteristics. Viral and host signals can induce a lymphatic transcriptional program, while the anatomical site and inflammatory environment further shape phenotype. This plasticity is relevant to immunotherapy because antigen processing, interferon responsiveness, adhesion molecules, and leukocyte trafficking differ across endothelial states. A cutaneous plaque with abundant perivascular lymphocytes may be immunologically distinct from a fibrotic nodule or a lymph-node lesion despite sharing HHV-8 positivity. Sampling site should therefore be recorded and, where feasible, the same lesion type should be followed longitudinally.

The early infected cell is not necessarily a fully transformed clone. KSHV promotes survival, proliferation, angiogenesis, and resistance to cellular stress, while paracrine signals recruit uninfected endothelial cells, fibroblasts, monocytes, and lymphocytes. This cellular admixture helps explain why changes in color, edema, or inflammatory infiltrate may occur rapidly after therapy even before the total number of infected spindle cells changes. It also complicates molecular analysis: bulk RNA sequencing can overestimate immune activation when a specimen contains many infiltrating leukocytes and underestimate tumor-intrinsic immune escape when infected cells are scarce. Digital pathology, cell-type deconvolution, single-cell methods, and spatial assays are consequently more informative than undissected bulk measurements alone.

### 3.2 Viral latency, lytic reactivation, and antigen availability

During latency, only a restricted set of viral genes is expressed. LANA tethers the viral episome to host chromatin, supports genome persistence during cell division, and alters pathways governing p53, retinoblastoma proteins, transcription, and epigenetic control. Viral cyclin, vFLIP, kaposins, and viral microRNAs contribute to survival and inflammatory signaling. Restricted latency is advantageous to the virus because it limits the number of antigens visible to immune surveillance. Nevertheless, incomplete latency, abortive lytic activity, and microenvironmental reactivation can expose additional antigens and release viral products that affect neighboring cells. The balance is dynamic rather than binary and may differ between lesions within one patient.

Lytic reactivation can be driven by hypoxia, oxidative stress, inflammation, epigenetic change, and therapeutic perturbation. It can increase antigen availability and potentially facilitate immune recognition, but it can also promote tumor growth through cytokines, chemokines, and angiogenic factors. This duality cautions against a simplistic assumption that more lytic replication is always favorable for checkpoint therapy (12, 13, 23). A high circulating viral load might identify antigen-rich disease, extensive tumor burden, a concurrent KSHV inflammatory syndrome, or ineffective host control. Predictive studies should therefore pair quantitative viral DNA with transcriptional or protein markers of latency and lytic activity, clinical disease burden, and treatment timing. The most informative endpoint may be the trajectory after therapy rather than the baseline value alone.

### 3.3 Innate immune evasion and inflammatory oncogenesis

KSHV has evolved multiple mechanisms to interfere with innate sensing, interferon production, and antiviral effector pathways. Viral proteins can attenuate pattern-recognition receptor signaling, manipulate ubiquitination, inhibit interferon-regulatory factors, and alter natural-killer-cell recognition. At the same time, infected cells and recruited leukocytes produce inflammatory mediators that support vascular proliferation and permeability. Viral interleukin-6, host IL-6, IL-10, vascular endothelial growth factor, and other mediators can create systemic and local manifestations that overlap with tumor progression (12, 24, 25). This inflammatory context may generate an apparently hot lesion that is nevertheless immunologically ineffective because effector cells are dysfunctional or spatially separated from infected spindle cells. Quantifying inflammation without defining its cellular source and functional state is therefore unlikely to yield a specific predictor.

Myeloid cells deserve particular attention. Tumor-associated macrophages can support angiogenesis, tissue remodeling, immunosuppressive cytokine production, and T-cell exclusion. Conventional M1/M2 categories are too coarse for KS, in which macrophages may simultaneously express inflammatory and suppressive programs. Spatial proximity between macrophage states, infected spindle cells, and CD8-positive lymphocytes may be more informative than total macrophage density (24–26). Neutrophil-rich inflammation, systemic lymphopenia, and high C-reactive protein may also reflect an unfavorable host-tumor relationship, but their predictive value for checkpoint blockade remains unknown. Prospective analyses should separate general inflammatory burden from a specific interferon-driven, antigen-presenting microenvironment that could support response.

### 3.4 Adaptive immunity, exhaustion, and checkpoint dependence

Adaptive control of KSHV depends on coordinated CD4-positive and CD8-positive T-cell responses as well as antibody and innate-cell functions. HIV provides a clear model in which loss of immune competence permits KS, but HIV-negative disease demonstrates that subtler or qualitatively different immune defects can be sufficient. Aging can reduce naive T-cell diversity and alter memory compartments. Pharmacological immunosuppression can selectively impair T-cell activation or proliferation. Chronic antigen exposure can produce progressive exhaustion characterized by PD-1 and other inhibitory receptors, altered metabolism, and reduced cytokine production. A reduced peripheral CD4 count may capture only one component of this biology and does not reveal whether tumor-infiltrating cells retain proliferative potential after PD-1 blockade.

Checkpoint dependence is best conceptualized as a state in which an otherwise competent antitumor or antiviral response is restrained by reversible inhibitory signaling. If immune failure is instead caused by absent antigen presentation, profound repertoire loss, fixed epigenetic exhaustion, dominant suppressive myeloid cells, or lack of T-cell entry, PD-1 monotherapy may be insufficient. Combination approaches could theoretically address these barriers through antiangiogenic normalization, immunomodulatory drugs, therapeutic vaccination, cytokine support, innate agonists, or agents that alter viral transcription. Each combination also introduces toxicity and mechanistic complexity. Biomarker studies should therefore identify the limiting step in the cancer-immunity cycle rather than merely count PD-1-positive cells.

### 3.5 Angiogenesis, hypoxia, and opportunities for combination therapy

KS is intrinsically angiogenic. VEGF signaling, hypoxia-inducible pathways, vascular leakage, and disordered lymphatic function contribute to lesion growth and edema. Abnormal vasculature can restrict effective lymphocyte trafficking and generate hypoxic niches that favor suppressive cells and dysfunctional metabolism. Antiangiogenic treatment may normalize vascular function, reduce edema, and facilitate immune-cell entry, providing a rationale for combinations with checkpoint inhibition. However, antiangiogenic activity can itself shrink or palliate lesions, making it difficult to attribute response in a single-arm combination study. The withdrawn pembrolizumab-lenvatinib trial illustrates both the scientific interest and practical difficulty of studying such combinations in a rare cancer. Future designs should incorporate a clear monotherapy benchmark, serial tissue or imaging evidence of vascular modulation, and toxicity monitoring for hypertension, bleeding, thrombosis, impaired wound healing, and cardiac effects.

Taken together, these biological observations provide a rationale for testing checkpoint blockade and for collecting candidate viral, tumor, microenvironmental, host, and on-treatment measurements prospectively. They do not establish clinical benefit, explain response in an individual patient, or justify treatment selection without comparative evidence and external validation.

Edema, elevated D-dimer, hypoalbuminemia, and extensive lesions can reflect tumor-associated vascular dysfunction, inflammation, nutritional reserve, treatment effects, and other comorbid processes. These variables should not be retroactively labeled predictive biomarkers. They may nevertheless influence trial eligibility, pharmacokinetics, toxicity, and interpretation of symptomatic improvement. A rigorous immunotherapy study should document baseline edema using standardized measurements, separate antitumor response from fluid shifts, and record anticoagulation and cardiovascular risk.

## 4 Clinical efficacy of checkpoint blockade

### 4.1 Pembrolizumab in classic and endemic KS

The principal prospective HIV-negative dataset is the multicenter, single-arm phase II trial NCT03469804. Seventeen adults with progressive classic or endemic KS requiring systemic treatment received pembrolizumab 200 mg every three weeks for eight cycles. Using modified AIDS Clinical Trials Group criteria, two patients achieved complete response and ten achieved partial response, yielding a best overall response rate of 71% (95% confidence interval, 44%-90%) within six months. Five patients had stable disease. Median follow-up was 20.4 months. Treatment-related adverse events occurred in 76%, with two grade 3 events and no treatment-related deaths (27). This is the most informative direct prospective signal, but it should be interpreted as proof of concept only: the small cohort, absence of a comparator, wide confidence interval, and pooled classic/endemic population preclude conclusions about comparative effectiveness, optimal sequencing, or biomarker-defined benefit.

### 4.2 Other checkpoint strategies and contextual evidence

Case reports support the possibility of deep or durable responses in selected classic KS, including aggressive PD-L1-positive disease, but publication bias is unavoidable (28, 29). A phase II study of nivolumab plus ipilimumab in classical KS was registered, yet no peer-reviewed outcome was identified in the present search. A planned pembrolizumab-lenvatinib study was withdrawn with zero enrollment. Pomalidomide plus nivolumab remains under investigation in virus-associated malignancies with or without HIV (14, 30, 31). In HIV-associated KS, checkpoint-inhibitor experience has shown responses and safety signals that strengthen biological plausibility, but these observations cannot be numerically merged with HIV-negative cohorts (32–34).

### 4.3 Interpreting response magnitude and durability

The 71% response proportion observed with pembrolizumab is hypothesis generating rather than practice defining. A 17-patient, single-arm trial cannot resolve differences across subtypes, prior therapies, disease extent, host context, or biomarker categories, and selection effects prevent an estimate of benefit relative to an alternative treatment. Modified ACTG criteria are appropriate for KS but differ from RECIST-based assessment; flattening, fading, lesion-number reduction, and edema improvement do not map directly to conventional radiographic tumor shrinkage. Cross-study comparisons should therefore report the criteria, assessment schedule, symptom outcomes, and duration of response rather than treat objective response proportions as directly interchangeable.

Duration is as important as initial response. Checkpoint therapy can generate long immune-mediated control after treatment cessation, but follow-up must be long enough to distinguish durable remission from temporary improvement. Future reports should provide swimmer plots or individual timelines showing prior treatment, treatment exposure, best response, time to response, duration of response, reason for discontinuation, subsequent therapy, and survival. Complete response should be confirmed and should account for noncutaneous disease. Stable disease may be meaningful in rapidly progressive KS but less informative in indolent classic disease. A prespecified clinical-benefit endpoint could combine durable response and prolonged stable disease, but its time threshold should be justified and sensitivity analyses should separate its components.

### 4.4 Pseudoprogression, inflammatory reactions, and response confirmation

Checkpoint inhibition can increase immune infiltration, granulomatous inflammation, or edema, potentially mimicking progression. In KS, where lesions are already inflammatory and vascular, the distinction may be particularly difficult. New discoloration, transient swelling, or enlargement of lymph nodes should be interpreted alongside symptoms, lesion photography, imaging, viral markers, and, when safe, biopsy. The granulomatous reaction reported in the phase II pembrolizumab study illustrates that immune activation can create clinically consequential findings unrelated to viable tumor expansion. At the same time, presumed pseudoprogression should not become a rationale for continuing ineffective therapy in a patient with worsening organ function or rapidly progressive visceral disease. Trials need explicit confirmation rules and criteria for treatment beyond initial progression.

Standardized photography should use consistent lighting, distance, camera settings, anatomical positioning, and a measurement scale. Index lesions should be selected prospectively, and lesion color and elevation should be recorded in addition to bidimensional measurements. Edema should be assessed independently. For nodal and visceral disease, cross-sectional imaging and organ-specific investigations are required, while PET uptake should be interpreted cautiously because inflammation can be metabolically active. Central blinded review may be unrealistic for every rare-disease study, but a central adjudication panel for ambiguous responses and suspected pseudoprogression would substantially improve reliability.

### 4.5 Safety interpretation in a rare and heterogeneous population

Safety cannot be inferred solely from the absence of treatment-related deaths in a small cohort. Classic KS patients may be older and have cardiovascular, endocrine, renal, or pulmonary comorbidity that modifies the consequences of immune-related adverse events. Endemic disease may occur in younger individuals but in settings with different infection burdens and access to subspecialty care. Iatrogenic KS presents the additional problem that immune activation may aggravate the underlying autoimmune disease or precipitate allograft rejection. The denominator for each safety analysis must be explicit, and attribution should distinguish immune-related events from infection, tumor inflammation, chemotherapy effects, and pre-existing disease. Time at risk continues after the last dose because delayed immune toxicity can occur.

Baseline evaluation should include medication history, prior autoimmune disease, transplantation, viral hepatitis, tuberculosis risk where epidemiologically appropriate, endocrine function, organ reserve, and an assessment of active infection. During treatment, laboratory and symptom monitoring should be adapted to the regimen and comorbidity profile. For HBV, HBsAg, anti-HBc, HBV DNA, alanine aminotransferase, and antiviral therapy should be documented. For transplant recipients, organ type, time since transplant, rejection history, immunosuppressive regimen, donor-specific antibodies where relevant, and graft function are essential. These variables are not merely exclusions; they define clinically important subgroups in which benefit-risk relationships may differ substantially from those in the published pembrolizumab cohort.

### 4.6 Positioning immunotherapy within systemic treatment

The available evidence does not establish checkpoint blockade as a universal first-line treatment for HIV-negative KS. Treatment selection begins with subtype, pace, anatomical extent, symptoms, reversibility of immune suppression, comorbidity, and patient preference. Limited cutaneous disease may be managed with local surgery, radiotherapy, intralesional treatment, or observation, depending on trajectory and burden. In iatrogenic disease, reduction or modification of immune suppression can be the most direct intervention. Disseminated, painful, ulcerated, edema-producing, rapidly progressive, nodal, or visceral disease more often requires systemic therapy (5, 7, 35). Liposomal anthracyclines and paclitaxel have established activity, whereas interferon, immunomodulatory drugs, and antiangiogenic approaches may be considered according to context and availability (8, 9, 11). The pembrolizumab phase II results support checkpoint inhibition as a plausible systemic option or clinical-trial strategy, but comparative sequencing against active chemotherapy has not been defined.

The desired treatment profile varies by clinical setting. Cytotoxic therapy can produce relatively rapid control, which may be important when edema compromises mobility or visceral disease threatens organ function. Checkpoint responses may be durable but can take time, and inflammatory changes can initially complicate assessment. Older patients may wish to avoid cumulative cytotoxic exposure yet may also have comorbidities that increase the consequences of immune-related endocrinopathy, pneumonitis, myocarditis, or other toxicity (36–38). A person with indolent classic KS and slowly increasing skin lesions presents a different decision from a younger patient with aggressive nodal or visceral disease (39–41). These differences should be visible in trial eligibility and subgroup reporting rather than absorbed into one pooled response estimate (42–44).

Treatment sequencing also affects biomarker interpretation. Prior chemotherapy can alter lymphocyte counts, macrophage composition, vascularity, and tumor-cell fraction. Radiotherapy can produce local immune and stromal changes. Antiangiogenic treatment can modify edema and leukocyte trafficking. Immunosuppressive drugs can suppress the pathway that checkpoint treatment is intended to restore. An archival pretreatment biopsy may therefore be biologically remote from the lesion exposed to immunotherapy. Studies should define a washout period, document intervening therapy, and obtain a new baseline specimen when feasible. Analyses should test whether prior systemic exposure modifies response, while recognizing limited power.

Until randomized evidence becomes available, treatment decisions should be shared and transparent. The clinician should explain the strength and limitations of the checkpoint evidence, alternative active therapies, expected timing of benefit, uncertainty regarding predictive biomarkers, and the need for toxicity surveillance. Enrollment in a prospective trial or registry is preferable when accessible because systematic follow-up can advance the field. Off-label use should still employ standardized lesion documentation, modified ACTG assessment, longitudinal safety monitoring, and complete reporting. A multidisciplinary team involving dermatology, oncology, pathology, infectious diseases or hepatology, and relevant transplant specialists can address the distinctive viral, oncological, and immune dimensions of HIV-negative KS.

## 5 Registered clinical trials

**Table 2** summarizes checkpoint-based or directly relevant immunotherapy trials identified in ClinicalTrials.gov. Registry entries reflect study status on July 25, 2026; recruitment status and planned enrollment may subsequently change (31).

**Table 2:** Selected registered clinical trials relevant to immune-based treatment of Kaposi sarcoma.

Trial	Population/design	Intervention	Status; N	Primary endpoint	Results/relevance
NCT03469804	Classic or endemic KS; multicenter, single-arm phase II	Pembrolizumab 200 mg every 3 weeks, 8 cycles	Registry: unknown; N = 17	Best overall response at 6 months	Published ORR 71% (CR 12%, PR 59%); median follow-up 20.4 months. Direct HIV-negative evidence. Limitations: small single-arm cohort; classic and endemic subtypes pooled.
NCT03219671	Classical KS; single-arm phase II	Nivolumab plus ipilimumab	Registry: unknown; planned N = 20	ORR at 6 months	No peer-reviewed outcome identified in the present search; should not be interpreted as efficacy evidence. Limitations: no peer-reviewed efficacy outcome available.
NCT05846724	Previously treated recurrent classic KS; single-arm phase II	Pembrolizumab plus lenvatinib	Withdrawn; actual N = 0	ORR at 6 months	No participants enrolled; documents interest in checkpoint/antiangiogenic combinations but provides no clinical evidence. Limitations: withdrawn before enrollment; no efficacy estimate.
NCT04902443	Virus-associated malignancies with or without HIV, including KS; phase I	Pomalidomide plus nivolumab	Recruiting; planned N = 58	Safety and tolerability	Potentially informs immunomodulatory/checkpoint combinations; KS- and HIV-specific outcomes are pending. Limitations: outcomes are pending and KS-specific results are not available.
NCT06638931	Advanced rare solid tumors including KS; single-arm phase II basket	Nivolumab; PD-L1 CPS >=10 required	Recruiting; planned N = 28	Efficacy at 2 years	Eligibility enriches for PD-L1-positive disease and limits inference to unselected KS. Limitations: PD-L1-enriched basket design; not generalizable to unselected KS.
NCT02595866	People with HIV and advanced cancers including KS; phase I	Pembrolizumab plus antiretroviral therapy	Completed; N = 58 overall	Safety and immune-related events	Contextual evidence for checkpoint safety in HIV, not direct HIV-negative KS evidence. Limitations: HIV-associated contextual study, not direct HIV-negative evidence.

The direct HIV-negative efficacy dataset is a small, single-arm study; records without peer-reviewed outcomes, those still recruiting, and withdrawn studies are included only to describe the development landscape. CPS, combined positive score; CR, complete response; KS, Kaposi sarcoma; ORR, objective response rate; PR, partial response. Source: ClinicalTrials.gov API v2, accessed July 25, 2026.

### 5.1 Lessons from the clinical-development landscape

The registry landscape reveals both scientific diversification and operational fragility. Pembrolizumab monotherapy reached a completed proof-of-concept study and produced the most persuasive HIV-negative evidence. By contrast, the nivolumab-ipilimumab registry record has not yielded an identifiable peer-reviewed outcome in the present search, and the pembrolizumab-lenvatinib study was withdrawn before enrollment. These outcomes are informative even though they do not estimate efficacy. Rare cancers face slow recruitment, geographically dispersed patients, competition with accepted off-label treatments, and limited commercial incentive. A trial may also exclude the patients most in need of new therapy through restrictive performance-status, organ-function, or immune criteria. International collaboration and pragmatic eligibility are therefore methodological requirements rather than administrative conveniences.

Basket trials can improve feasibility but introduce interpretive challenges. A trial enrolling many rare solid tumors may include only one or two patients with KS, and a tumor-agnostic eligibility rule such as PD-L1 combined positive score of at least 10 can create selection that is biologically unvalidated in KS. Overall basket efficacy cannot be transferred to the KS subset. Protocols should prespecify a minimum KS sample, subtype and HIV-status reporting, KS-specific response assessment, and separate estimates even when formal hypothesis testing is impossible. Virus-associated malignancy baskets are conceptually attractive because they can share immune and viral assays, yet KSHV-associated lymphoma, KS, EBV-driven tumors, and HPV-related cancers have distinct target cells and microenvironments. Hierarchical analysis can borrow strength without assuming identical treatment effects.

Combination trials should be built around a mechanistic hypothesis and feasible correlative endpoints. Pomalidomide can modulate immune function and has direct activity in KS, so a pomalidomide-nivolumab response cannot be attributed automatically to checkpoint blockade. The trial should evaluate whether the combination improves depth or duration beyond expected pomalidomide activity and whether immune changes support synergy. Sequential or randomized designs are preferable when feasible. In a small multi-cohort platform, adaptive allocation could discontinue inactive combinations while expanding promising subtype-specific cohorts. Centralized assays and a common data dictionary would permit pooled translational analyses even when treatment modules differ.

## 6 Predictive biomarkers

### 6.1 Viral biomarkers

Circulating KSHV DNA is an attractive biomarker because it is disease specific, repeatable, and potentially responsive to treatment. However, assay matrices, extraction methods, targets, and reporting units vary (45–47). Detectable DNA may reflect tumor burden, lytic replication, or concurrent KSHV inflammatory disease rather than checkpoint sensitivity itself (48–50). Baseline value, early change, and clearance should therefore be analyzed separately. Tissue LANA is essential diagnostically, but semiquantitative LANA intensity has not been prospectively validated as a response predictor. A more informative approach may quantify infected-cell burden together with latent and lytic transcriptional programs (23, 51, 52).

### 6.2 Tumor and microenvironmental biomarkers

PD-L1 is biologically relevant but analytically fragile. Antibody clone, scoring compartment, cutoff, lesion age, and prior treatment can alter classification. Moreover, reported responses in PD-L1-negative KS indicate limited negative predictive value (29, 51, 53). Likewise, low tumor mutational burden does not imply low antigenicity in a virus-associated cancer. Multiplex immunohistochemistry, spatial transcriptomics, and single-cell profiling could better distinguish inflamed, exhausted, immune-excluded, and myeloid-dominant lesions. Candidate signatures should include CD8 density, PD-1 and other exhaustion markers, regulatory T cells, macrophage state, interferon-response genes, antigen-presentation machinery, and vascular-stromal organization.

### 6.3 Host and dynamic biomarkers

HIV seronegativity is an etiological descriptor, not a complete immune phenotype. Age-related immune remodeling, primary immune defects, malignancy-associated lymphopenia, transplantation, immunosuppressive drugs, chronic viral coinfection, malnutrition, and systemic inflammation may all modify checkpoint activity and toxicity (36, 54, 55). CD4 count and CD4/CD8 ratio should be captured as continuous variables rather than arbitrary eligibility thresholds. On-treatment measurements are particularly important: early reduction in KSHV DNA, expansion of virus-reactive T-cell clones, conversion from immune-excluded to inflamed tissue, or coordinated cytokine changes may prove more predictive than a single baseline biopsy (49, 56, 57).

### 6.4 Analytical requirements for KSHV DNA

Before KSHV DNA can be tested as a predictive biomarker, the analytical question must be specified. Plasma primarily contains cell-free DNA, whereas whole blood and peripheral-blood mononuclear cells include cell-associated virus. Serum can differ from plasma because clotting releases cellular material. Laboratories use different extraction volumes, target genes, standards, lower limits of quantification, and reporting units. A result described simply as positive or negative is therefore difficult to compare across centers. Prospective trials should select one primary matrix, report preanalytical handling and time to processing, use a calibrated quantitative assay, and retain aliquots for bridging studies. Parallel plasma and cellular measurements in an initial cohort could determine which compartment best associates with disease and treatment response.

The statistical representation of viral load also matters. Values may be undetectable, intermittently detectable, or highly skewed. Analyses should predefine handling below the limit of detection, use log transformation where appropriate, and avoid selecting an optimal cutoff from a small dataset. Baseline viral load should be modeled alongside measured disease burden to determine whether it adds information beyond stage. Early change could be expressed as absolute decline, log-fold change, clearance, or area under the longitudinal curve. A landmark time must be chosen before outcome evaluation to avoid immortal-time bias. If treatment induces lytic activity before immune clearance, an early transient increase could have a different meaning from persistent elevation; dense sampling in pilot studies can establish the relevant kinetics.

### 6.5 PD-L1 assay interpretation

PD-L1 is not a single reproducible biological quantity. Tumor proportion score, combined positive score, and immune-cell scores answer different questions, and KS spindle cells can be difficult to distinguish from endothelial and inflammatory cells on routine staining. Clone-specific sensitivity, fixation, decalcification, old archival blocks, and focal expression introduce further variability. Digital image analysis may improve reproducibility only if the cellular compartment is accurately segmented and HHV-8-positive spindle cells can be identified. Dual or multiplex staining that combines LANA, endothelial markers, PD-L1, and immune-cell markers could attribute expression more reliably than a single chromogenic stain (29, 51).

From a clinical perspective, PD-L1 should initially be treated as a continuous and spatially resolved exploratory variable. Reporting only a positive/negative category discards information and invites unstable thresholds. Studies should present response by the full distribution, test prespecified cutoffs only when justified and evaluate whether PD-L1 adds discrimination to clinical and viral variables. A predictive effect requires evidence that the association differs according to treatment, ideally in a randomized comparison. In a single-arm study, PD-L1 can be associated with response but cannot distinguish treatment prediction from a generally favorable immune microenvironment. The documented possibility of response despite negative staining makes clear that PD-L1 negativity should not currently deny an otherwise appropriate patient access to a clinical trial.

### 6.6 Genomic and transcriptomic biomarkers

Tumor mutational burden has become a familiar pan-cancer biomarker, but its biological relevance in KS is limited by viral oncogenesis, low neoplastic cellularity, and technical challenges in sequencing small or inflamed lesions. Low TMB does not quantify viral antigen diversity or immune recognition. MSI-high disease is expected to be uncommon and should be reported when tested, but its rarity limits population-level utility. Genomic profiling may nevertheless identify host alterations affecting antigen presentation, interferon pathways, chromatin regulation, or oncogenic signaling. Such findings are hypothesis generating unless recurrent and functionally validated. Tumor-only sequencing also risks misclassifying germline variants, particularly in younger or atypical cases where inherited immune susceptibility may be relevant (21, 53).

Transcriptomic approaches can capture interferon signaling, cytotoxic lymphocytes, antigen presentation, endothelial state, hypoxia, myeloid suppression, and latent or lytic viral programs simultaneously. However, a signature trained in common epithelial cancers should not be assumed valid in KS. A KS-specific signature requires careful normalization, cell-composition adjustment, locked gene weights, and an assay deployable on routine tissue. Single-cell RNA sequencing can identify cellular states but is sensitive to tissue dissociation and may lose fragile endothelial populations. Spatial transcriptomics preserves architecture but has cost, resolution, and batch limitations. A rational development sequence is discovery with high-dimensional methods, reduction to a robust multiplex panel, and validation using a prespecified scoring algorithm.

### 6.7 Immune repertoire and functional assays

T-cell receptor sequencing can determine clonality, expansion, and overlap between blood and tumor. Expansion of pre-existing intratumoral clones after PD-1 blockade could support pharmacodynamic activity, whereas recruitment of new clones might indicate epitope spreading. Yet clonality is not inherently beneficial: a highly clonal population may be exhausted, unrelated to KSHV, or driven by another chronic infection. Pairing repertoire data with functional stimulation using validated KSHV antigens, cytokine production, activation markers, or multimer assays would improve specificity. These methods require standardized antigens and HLA-aware interpretation and may not be feasible as routine companion diagnostics. They are nevertheless valuable in early translational studies that seek to establish whether response is mediated predominantly by virus-specific or broader tumor-reactive immunity (26, 53).

Peripheral immune phenotyping should extend beyond CD4 and CD8 counts. Naive, central-memory, effector-memory, regulatory, activated, proliferating, and exhausted subsets can be measured by flow or mass cytometry. Myeloid and natural-killer compartments are also relevant. High-dimensional panels must be designed prospectively, with stable sample processing, viability thresholds, batch controls, and blinded analysis. Because blood may not reflect the lesion, paired tissue measurements are critical. The primary aim should not be to test every marker independently but to identify a small number of biologically coherent features that improve a prespecified model.

### 6.8 Composite models and clinical utility

A composite model is justified when no single marker captures the necessary biology. One possible structure includes a viral-activity score, an inflamed-tumor score, a host-immune-reserve score, and an early dynamic-response score. Clinical subtype and prior therapy should remain explicit covariates rather than being hidden inside a molecular classifier. Model development must respect the ratio of events to candidate parameters. Penalized regression, Bayesian shrinkage, or dimension reduction can reduce overfitting, but no statistical method rescues a dataset with too few informative outcomes. Internal validation should quantify optimism, calibration, discrimination, and decision-curve performance. Reporting only an area under the receiver-operating-characteristic curve is inadequate.

Clinical utility requires a defined decision. A model could enrich a trial for likely responders, identify patients who need combination therapy, recommend an early switch for molecular nonresponse, or intensify toxicity monitoring. Each use has different acceptable false-positive and false-negative rates. A biomarker that modestly enriches response may be useful for a toxic combination but inappropriate for excluding patients from a relatively tolerable monotherapy. Decision thresholds should therefore be selected using clinical consequences, not statistical convenience. After external validation, an impact study should test whether biomarker-guided care improves outcomes compared with standard clinical judgment. Until that sequence is completed, the multidomain framework should be described as investigational. The proposed biomarker-development pathway is summarized in **Figure 2**.

**Figure 2:**
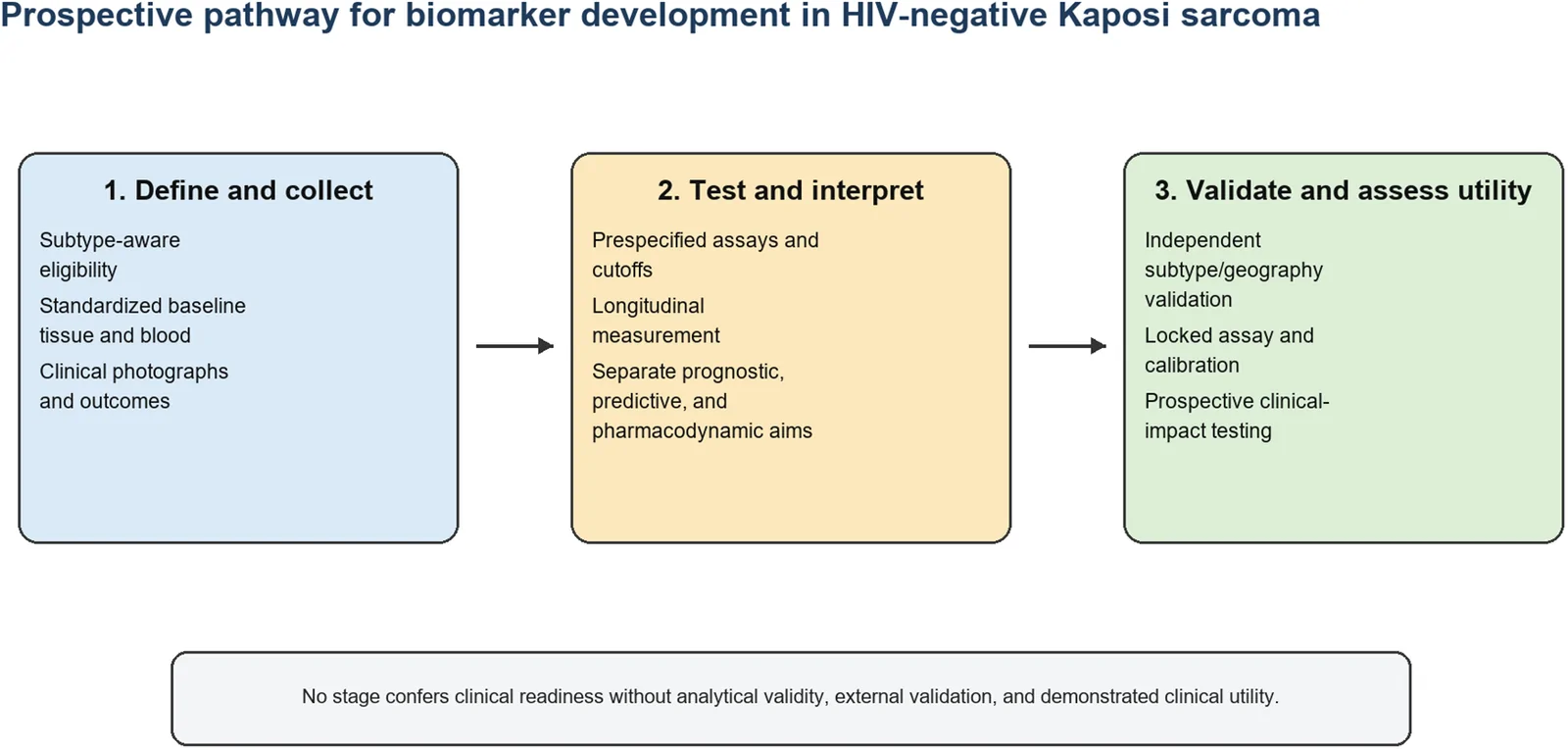
Simplified author-created prospective pathway for biomarker development in HIV-negative Kaposi sarcoma. Phase 1 defines a subtype-aware cohort and standardized baseline sampling; phase 2 applies prespecified assays and longitudinal outcomes while separating prognostic, predictive, and pharmacodynamic aims; phase 3 requires independent validation, a locked assay, calibration, and clinical-utility assessment. No stage establishes clinical readiness without analytical validity, external validation, and demonstrated clinical utility.

### 6.9 Key messages and interpretation

What is known: pembrolizumab has shown an encouraging signal in a small, single-arm prospective cohort of classic or endemic KS. What remains uncertain: comparative effectiveness, sequencing, subtype-specific activity, and the safety and efficacy of combinations. What is biologically plausible but unproven: that viral state, immune contexture, host factors, or early on-treatment changes identify checkpoint benefit. What requires prospective validation: reproducible, prespecified assays tested in subtype-aware cohorts with appropriate comparator evidence and independent external validation. Candidate biomarkers and their recommended research use are summarized in **Table 3**.

**Table 3:** Candidate biomarkers for immune-checkpoint therapy in HIV-negative Kaposi sarcoma.

Domain	Candidate marker	Biological rationale	Current evidence	Recommended use
Viral	Circulating KSHV/HHV-8 DNA	Approximates systemic viral activity and, imperfectly, disease burden	Prognostic/disease-activity evidence; predictive validation lacking	Serial exploratory marker at baseline and early treatment
Viral	Tissue LANA and latent/lytic state	Captures infected-cell burden and antigenic state	Strong diagnostic rationale; limited treatment-predictive data	Standardize digital scoring and paired biopsies
Tumor	PD-L1 expression	May mark adaptive immune resistance	Responses can occur despite PD-L1 negativity; assay and cutoff heterogeneity	Do not use as a stand-alone exclusion marker
Tumor	TMB/MSI	Conventional pan-cancer predictors of checkpoint response	KS is commonly low-TMB; virus-derived antigens may dominate	Exploratory only
Microenvironment	CD8 infiltration/exhaustion and spatial phenotype	Measures available antitumor/antiviral effector cells and inhibitory signaling	Mechanistically compelling; prospective validation needed	Multiplex IHC or spatial transcriptomics
Host	CD4 count and CD4/CD8 ratio	Reflects systemic immune competence independent of HIV serostatus	Potential modifier; not validated as a response cutoff	Record continuously, not as a binary eligibility rule
Dynamic	Early KSHV-DNA and immune-profile change	On-treatment change may outperform static baseline markers	Hypothesis-generating	Pre-specify landmark analyses in prospective trials

None has sufficient prospective validation to serve as a stand-alone companion diagnostic or to guide routine treatment selection.

## 7 Practical clinical and translational workflow for HIV-negative KS

The corresponding clinical and translational workflow is summarized in **Figure 3**. This section provides a practice-oriented and research-oriented workflow rather than a case report. It is designed to prevent HIV seronegativity from being mistaken for a complete assessment of immune competence, while avoiding the unsupported inference that routine clinical variables predict benefit from checkpoint blockade.

**Figure 3:**
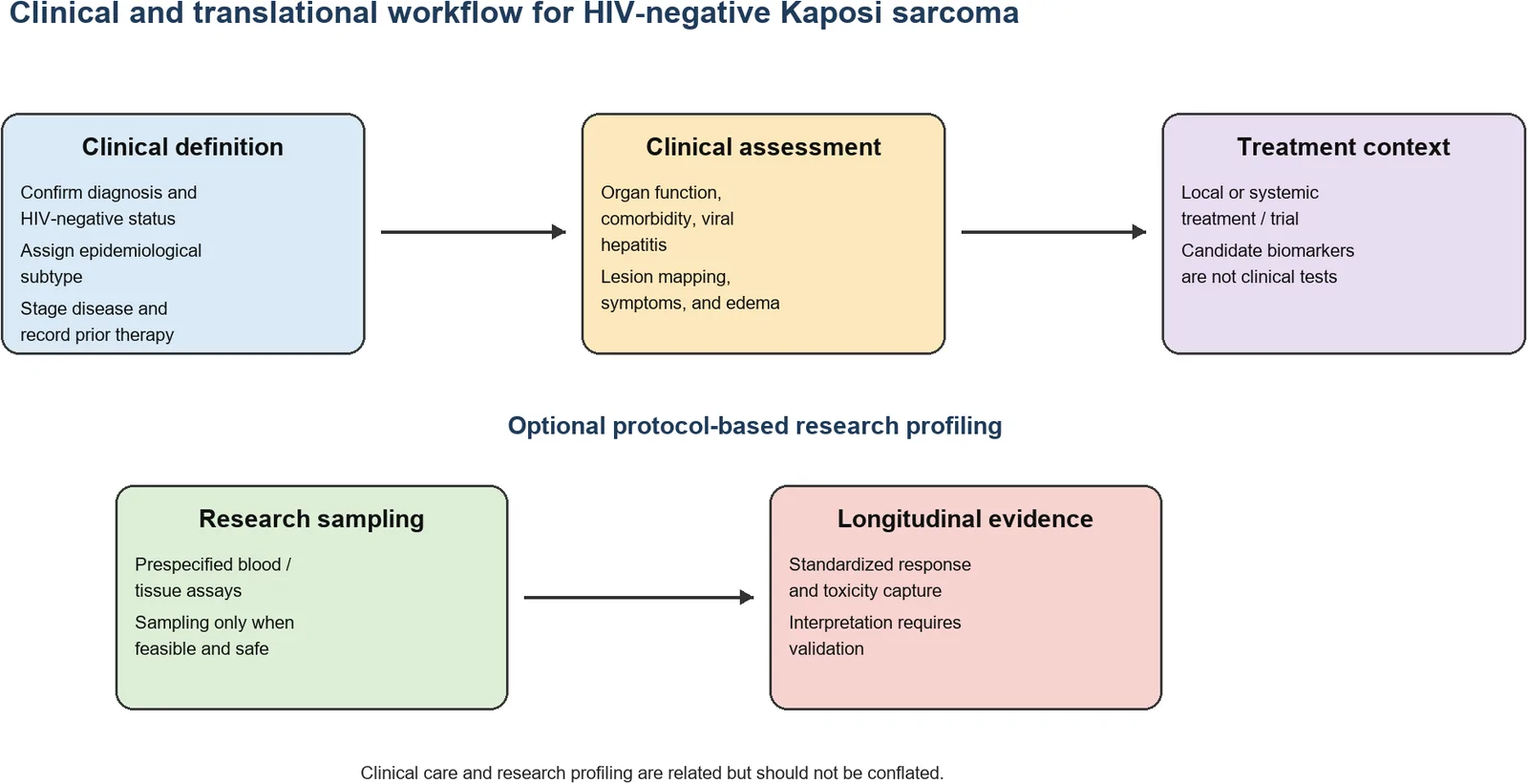
Simplified author-created clinical and translational workflow for HIV-negative Kaposi sarcoma. The upper pathway separates clinical definition, assessment, and treatment context. The lower pathway describes optional protocol-based research profiling, longitudinal evidence capture, and cautious interpretation. The framework clarifies that clinical care and exploratory biomarker research are related but should not be conflated, and it is not a validated clinical decision algorithm.

Direct HIV-negative evidence for checkpoint inhibition remains limited, and the available literature does not establish PD-L1, tumor mutational burden, circulating KSHV DNA, CD4 count, or inflammatory markers as validated treatment-selection biomarkers. These measurements may be clinically informative or scientifically useful, but they should be interpreted according to their analytical limitations and the question being asked: diagnosis, prognostic stratification, pharmacodynamic monitoring, or treatment-effect modification.

The workflow below is therefore a proposed theoretical roadmap for future prospective studies. It does not recommend a specific checkpoint regimen for any individual and does not substitute for subtype-specific clinical assessment, standard systemic-treatment options, or trial eligibility criteria.

### 7.1 Diagnostic confirmation and subtype assignment

The first step is diagnostic and epidemiological confirmation. Pathology should verify characteristic morphology and HHV-8/LANA positivity, ideally with central review in a prospective study. Mimics such as other vascular neoplasms and reactive proliferations should be excluded using morphology and an appropriate immunohistochemical panel. HIV testing should be current and documented, but evaluation should proceed to subtype assignment rather than stop at a negative result.

History should address country and region of origin, ancestry where relevant, KSHV exposure context, prior transplantation, autoimmune disease, corticosteroids, biologic agents, chemotherapy, recurrent infections, and constitutional symptoms suggesting concurrent KSHV-associated inflammatory or lymphoproliferative disease. Staging should distinguish skin, oral, nodal, pulmonary, gastrointestinal, bone, and other visceral involvement and should document functional impairment and edema.

### 7.2 Baseline assessment, treatment context, and research profiling

Baseline treatment assessment should include blood count, renal and hepatic function, albumin, endocrine tests where relevant, glucose, cardiopulmonary risk, concomitant medicines, and performance status. Viral hepatitis testing should guide prophylaxis and monitoring. For an HBsAg-positive patient, HBV DNA, liver function, and adherence to antiviral therapy are central. Active infection should be treated before immunotherapy when possible, and transplant recipients require a separate graft-rejection risk assessment (5, 36, 54).

Treatment selection begins with subtype, disease pace, anatomical extent, symptoms, reversibility of immunosuppression, comorbidity, and patient preference. Local treatment may be appropriate for limited disease. For extensive, symptomatic, edema-producing, rapidly progressive, nodal, or visceral disease, established systemic options should be considered alongside clinical trials (7, 8, 35). Current evidence does not define a universal first-line role or optimal sequencing for checkpoint blockade in HIV-negative KS (9–11).

Within an approved research protocol, a pre-specified panel may include quantitative KSHV DNA, immune-cell phenotyping, plasma proteins, and tissue assays. A core tissue panel can combine LANA, endothelial markers, PD-L1, CD3, CD8, FOXP3, macrophage markers, proliferation, and antigen-presentation proteins. High-dimensional spatial, transcriptomic, genomic, or T-cell-repertoire assays are discovery tools; their use should be linked to a defined hypothesis, assay quality control, and a plan for independent validation.

### 7.3 Longitudinal assessment and cautious interpretation

Baseline photography and lesion mapping should be repeated using the same protocol. Longitudinal blood sampling can be scheduled at pre-specified pharmacodynamic time points and at response or progression when feasible. A paired biopsy should answer a defined question, such as whether immune-cell entry or antigen-presentation features differ between responding and progressing lesions. Participant burden and clinical safety should govern the intensity of repeat sampling.

Interpretation should integrate clinical outcomes with laboratory and tissue findings rather than privilege a single marker. Concordant changes, such as standardized lesion improvement accompanied by a pre-specified biological change, can generate a testable hypothesis but do not establish prediction in an uncontrolled cohort. Candidate predictive claims require an appropriate comparator, treatment-by-marker interaction analysis where feasible, locked assay thresholds, and external validation.

## 8 Safety and special populations

The phase II pembrolizumab study suggests an acceptable but nontrivial toxicity profile. Immune-mediated pancreatitis, granulomatous inflammation, and cardiac decompensation require attention, particularly in older classic-KS populations with comorbidity (58, 59). For transplant-associated KS, checkpoint blockade may threaten graft function and must be evaluated separately from other HIV-negative disease. Chronic HBV is not an absolute biological contraindication, but antiviral therapy, baseline HBV DNA, and longitudinal monitoring are essential (35, 36, 54). Cytopenias, malnutrition, edema, and inflammatory syndromes may complicate attribution of toxicity and should be optimized or documented before treatment.

## 9 Implications for future trials and evidence gaps

The field is limited by small cohorts, subtype mixing, inconsistent staging and response criteria, incomplete duration-of-response reporting, and nonstandardized biomarker assays (6, 24, 26). Future multicenter studies should stratify classic, endemic, iatrogenic, and unclassified HIV-negative KS. Modified ACTG criteria should be supplemented by patient-reported symptoms, edema, lesion imaging, duration of response, progression-free survival, overall survival, and corticosteroid exposure (27, 31, 60). Central pathological review and prespecified rules for nodal or visceral disease are needed.

A practical translational design would collect blood and tissue at baseline, approximately six weeks, twelve weeks, and progression. Viral DNA, immune-cell subsets, cytokines, T-cell receptor clonality, multiplex tissue imaging, and transcriptomic signatures should be analyzed longitudinally. Given rarity and subgroup heterogeneity, Bayesian hierarchical models could borrow information across subtypes while preserving subtype-specific estimates. Biomarker claims should be tested through interaction analyses rather than simple correlations within responders. External validation and assay harmonization are prerequisites for clinical use.

### 9.1 Multicenter platform design and equitable implementation

A realistic development program would use an international multicenter platform with a common screening and biomarker protocol. Participants would enter subtype-defined cohorts for classic, endemic, iatrogenic nontransplant, transplant-associated, and unclassified HIV-negative KS. An initial checkpoint-monotherapy module could establish a reproducible benchmark in populations for whom treatment is ethically acceptable. Additional modules could evaluate rational combinations only after monotherapy activity and safety are characterized. Shared eligibility, response, imaging, photography, specimen, and adverse-event procedures would permit cross-module learning. HIV-associated KS could enter a parallel cohort but should be analyzed separately, with prespecified bridging analyses rather than simple pooling.

Eligibility should represent practice while protecting safety. Histological confirmation and a defined indication for systemic treatment are essential. Measurable cutaneous disease, evaluable nodal or visceral disease, or both should be specified. Performance-status and organ-function limits should allow abnormalities attributable to KS when medically manageable. Prior systemic therapy should be stratified rather than universally excluded. Patients with controlled HBV could participate with antiviral therapy and monitoring. Autoimmune and transplant cohorts require separate rules. Mandatory fresh tissue can improve science but impede recruitment; accepting archival tissue or fresh biopsy when safely obtainable may be more feasible than excluding patients without an accessible lesion.

### 9.2 Endpoints and estimands

The primary estimand should specify population, treatment condition, endpoint, intercurrent-event strategy, and summary measure. Objective response by modified ACTG criteria is practical for an early-phase study, but it should be complemented by duration of response and a landmark durable-response rate. Discontinuation for toxicity, initiation of alternative KS therapy, death, and loss to follow-up require prespecified handling. Patient-reported pain, mobility, edema-related function, appearance-related distress, and global quality of life can capture benefits that lesion counts miss. Visceral and nodal disease need compatible radiological definitions. Overall survival remains important but may be confounded by subsequent therapy and may require prolonged follow-up in indolent classic disease.

Biomarker endpoints should distinguish prognostic, predictive, and pharmacodynamic objectives. In a single-arm module, baseline features can be associated with response but should be called candidate response markers rather than definitively predictive. A randomized module enables a treatment-by-marker interaction, although power may be limited. Dynamic markers require a landmark population alive and under observation at sampling; analyses must account for this selection. Multiplicity should be controlled through a limited hierarchy of primary biomarker hypotheses, with remaining high-dimensional analyses labeled exploratory. Protocols and statistical analysis plans should be publicly available before database lock.

### 9.3 Statistical strategy for rare subtypes

Conventional subgroup analyses are unstable when each subtype contains few participants. A Bayesian hierarchical model can estimate cohort-specific response probabilities while allowing partial borrowing determined by observed similarity. Robust priors or mixture models can limit inappropriate borrowing when one cohort behaves differently. Decision thresholds for expansion or futility should be calibrated by simulation under plausible response and recruitment scenarios. Posterior probabilities and credible intervals should be presented with raw patient-level outcomes. Time-to-event analyses may use hierarchical survival models, but assumptions must be transparent and frequentist sensitivity analyses can assess dependence on prior choices.

Prediction-model development should be separated from treatment monitoring. A discovery set can prioritize coherent features, but a locked model requires an independent sample. If external validation cannot be completed within one trial, a prospective registry network could apply the locked assay across centers. Missing tissue and failed assays should be reported by reason; imputation is appropriate only when assumptions are credible. Calibration, Brier score, discrimination, and decision-curve analysis should accompany a classifier. Subgroup transportability is critical: a model developed predominantly in European classic KS should not be promoted for endemic African disease without external validation.

### 9.4 Resistance mechanisms and rational next studies

Primary resistance may reflect absent antigen presentation, insufficient effector-cell entry, dominant suppressive myeloid or regulatory populations, fixed T-cell dysfunction, or signaling pathways that allow survival despite immune attack. Acquired resistance after initial response may involve loss of recognized antigens, impaired interferon signaling, evolution of the infected-cell population, or compensatory checkpoints. Progression biopsy should be prioritized because pretreatment tissue alone cannot distinguish these mechanisms. Separate progressing and responding lesions within the same patient offer a particularly informative comparison while controlling for systemic host factors. Molecular observations should be validated functionally before motivating a combination trial.

Combination selection should address an identified barrier. Antiangiogenic therapy may improve lymphocyte entry; immunomodulatory agents may enhance T- and natural-killer-cell activity; therapeutic vaccination could increase KSHV-specific responses; innate agonists might improve antigen presentation; and selected viral-state interventions could alter antigen availability. The expected benefit must be balanced against additive toxicity and against activity of the partner agent alone. Randomized phase II modules, adaptive multi-arm platforms, or short mechanistic windows followed by combination therapy can produce more interpretable evidence than uncontrolled combination series.

### 9.5 Reporting and data-sharing standards

Clinical reports should provide patient-level subtype, age, sex, geography, immune-suppression context, disease sites, prior therapy, regimen, exposure, best response, response time, duration, discontinuation reason, toxicity, and follow-up. Biomarker reports should state specimen type, collection time, processing and storage conditions, platform, antibody clone, scoring method, cutoff provenance, quality-control failure, and blinding. Rare combinations of age, geography, and phenotype can be identifiable, so controlled-access repositories may be needed. Harmonized data dictionaries can preserve analytic value while protecting participants.

Prospective registration should include all cohorts and endpoints, and records should be updated when recruitment stops or results appear. Negative and inconclusive studies are especially important in a field vulnerable to exceptional-responder bias. Core outcome and specimen sets developed with patients, clinicians, pathologists, virologists, laboratory scientists, and statisticians would reduce waste. Because endemic KS is concentrated in settings where access to trial infrastructure, pathology support, longitudinal sampling, and high-dimensional assays may be constrained, future platforms should use geographically inclusive partnerships, feasible core assays, and harmonized but context-sensitive procedures. High-quality evidence will depend less on continually adding isolated markers than on consistent measurement, complete reporting, reproducibility, and independent validation across collaborative studies.

## 10 Conclusions

PD-1 blockade has shown an encouraging but uncertain signal in one small prospective cohort of classic and endemic HIV-negative KS; the evidence remains insufficient to establish comparative effectiveness, optimal sequencing, or a validated predictive biomarker. PD-L1 expression, tumor mutational burden, and KSHV DNA should not be used alone to select or exclude patients. Future progress depends on subtype-aware and geographically inclusive studies with standardized outcomes, reproducible assays, longitudinal sampling, transparent reporting, and independent validation. The proposed multidomain framework organizes research priorities rather than guiding routine clinical decisions.
